# Association Between Uric Acid and Worsening Peripheral Microangiopathy in Systemic Sclerosis

**DOI:** 10.3389/fmed.2021.806925

**Published:** 2021-12-24

**Authors:** Eleni Pagkopoulou, Stergios Soulaidopoulos, Eva Triantafyllidou, Afrodite Malliari, George D. Kitas, Alexandros Garyfallos, Theodoros Dimitroulas

**Affiliations:** ^1^Fourth Department of Internal Medicine, Hippokration Hospital, Aristotle University of Thessaloniki, Thessaloniki, Greece; ^2^First Department of Cardiology, Hippokration General Hospital, National and Kapodistrian University of Athens, Athens, Greece; ^3^International Hellenic University, Thessaloniki, Greece; ^4^Department of Rheumatology, Dudley Group of Hospitals, NS Foundation Trust, Dudley, United Kingdom; ^5^Arthritis Research UK Centre for Epidemiology, University of Manchester, Manchester, United Kingdom

**Keywords:** uric acid, nailfold video-capillaroscopy, microvasculopathy, systemic sclerosis, cardiovascular

## Abstract

**Objective:** The key element in the pathogenesis of systemic sclerosis (SSc) is microcirculatory changes in several vascular beds. Uric acid is associated with endothelial dysfunction and therefore, microvascular damage. The aim of this study was to examine the association between uric acid (UA) and peripheral microvascular involvement in patients with SSc.

**Methods:** We included consecutive, consenting patients with SSc. Serum UA, urea and creatinine were measured, and glomerular filtration rate (GFR) was calculated with CKD-EPI. All participants underwent nailfold video-capillaroscopy (NVC) to evaluate the microcirculation.

**Results:** A total of 64 patients (95.3% women) were included in the study. UA levels were significantly associated with the number of avascular areas (*r* = 0.290; *p* = 0.020), whereas no correlation was shown for the GFR (*r* = −0.065; *p* = 0.609). A significant trend of UA in the three capillaroscopic patterns was shown (3.90 ± 1.52 vs. 4.15 ± 0.98 vs. 5.38 ± 2.26; for early, active, and late patterns respectively, *p* = 0.028). Multivariate analysis showed that male gender (β = 3.049; 95% CI = 0.997–5.101) and UA (β = 0.352; 95% CI = 0.117–0.588) were independently associated with the number of avascular areas.

**Conclusion:** These data suggest that UA levels are significantly associated with the capillaroscopic patterns, reflecting a progressive microvasculopathy.

## Introduction

In systemic sclerosis (SSc), inflammation and microvascular dysfunction appear to be the main events that progressively stimulate fibrotic process. The precise etiology of fibrotic changes remains partially understood and may include impaired communication between endothelial cells, epithelial cells and fibroblasts, lymphocyte activation, autoantibody production, inflammation, and connective tissue fibrosis (1). Alterations in microvasculature are considered the hallmark of SSc vascular involvement and occur in the first stages of the disease. Given the heterogenicity of clinical symptoms and organ involvement, there is an ongoing effort to establish biomarkers for the evaluation of microvasculopathy (2), which represents one of the earliest clinical manifestations of SSc presented in various guises such as Raynaud's phenomenon, digital ulcers, and pulmonary arterial hypertension (PAH) (3).

Uric acid (UA) is the final oxidation product of purine metabolism. Elevated serum UA levels have been associated with endothelial dysfunction, possibly by decreasing nitric oxide availability (4) and stimulating vascular smooth muscle cell proliferation leading to arterial stiffness, and gradually, widespread microvascular damage (5–7). UA levels have been found elevated in SSc patients and have been associated with the presence of vascular complications including pulmonary arterial hypertension (PAH) (8) digital ulcers (9) and abnormal findings in nailfold video capillaroscopy (NVC) (10); the latter is a non-invasive and reproducible imaging technique of the capillary vascular bed, used for the assessment of peripheral microvascular damage in SSc. It is extensively used in the differentiation between primary and secondary Raynaud's phenomenon in daily practice. “Abnormal nailfold capillaries” (when referring to the “scleroderma pattern”) are included in the 2013 American College of Rheumatology (ACR)/ European League Against Rheumatism (EULAR) classification criteria for SSc (11). Cumulative data suggest that NVC measurements can serve as a reliable marker of the extent and severity of microvasculopathy in different vascular districts such as pulmonary (12) and myocardial microcirculation (13) to the point that NVC is currently considered as a surrogate marker of SSc progression (14). However, the association between UA and NVC changes in SSc has not been well-established.

In this context, the aim of this study was to investigate the potential relationship between UA levels and microvascular alterations assessed by NVC in a large well-characterized cohort of SSc patients.

## Materials and Methods

### Study Participants and Inclusion / Exclusion Criteria

The study included consecutive patients with SSc attending the Scleroderma Clinic of the Fourth Department of Internal Medicine, Hippokration General Hospital, Thessaloniki, Greece, between March 2018 and September 2020, who were screened for the study. All patients satisfied the revised EULAR/ACR criteria for the diagnosis of SSc (11). The exclusion criteria included past diagnosis of cardiovascular disease defined as coronary heart disease, stroke, or peripheral vascular disease, diabetes mellites, as well as patients with carotid artery surgical procedures. Patients on diuretics were also excluded. The study had ethics approval from the Ethics Committee of the School of Medicine, Aristotle University of Thessaloniki and written informed consent was obtained from all participants according to the Declaration of Helsinki.

### Protocol Overview

All participants underwent a thorough physical examination and demographic data were collected by a questionnaire. Complete medical history was also recorded which included the duration of the disease, existence of pulmonary hypertension, pulmonary fibrosis, or esophageal motility disorders, as documented by imaging or endoscopic examination, respectively, as well as medication and cardiovascular risk factors (smoking, hypertension).

### Parameters of Interest and Definitions

Various hematological and biochemical laboratory parameters such as routine biochemistry and hematology, lipid and bone profile tests, inflammatory markers such as erythrocyte sedimentation rate (ESR) and Creactive protein (CRP), immunological markers such as antinuclear antibodies (ANA), anti-centromeric antibodies (ACA), and anti-topoisomerase IIa (anti-scl-70) antibodies were tested. Serum UA and creatinine levels were measured with photometric measurement of the solution and GRF was calculated using the CKD-EPI equation (15). NVC assessment and blood sampling were performed the same day. Blood pressure was recorded according to 2018 ESC/ESH Guidelines for the management of arterial hypertension, with a validated oscillometric device (16). Arterial hypertension was defined on the basis of the patients' history (self-reposted hypertension) and/or antihypertensive medication intake.

### NVC Assessment

Study participants underwent NVC using an Optilia Digital Capillaroscope and a 200 × contact lens and the photos collected were analyzed with OptiPix Capillaroscopy (Sollentuna, Sweden) software system. Prior to performing the test, patients were placed in a quiet environment at a temperature between 20 and 25°C. A drop of cedar oil was placed on each fingernail prior to observation for better image analysis. The findings were classified in one of the following qualitative patterns: early, active, and late NVC pattern (17). The “early” pattern was characterized by a few enlarged or giant capillaries and relatively well-preserved capillary distribution; the “active” pattern was characterized by numerous giant capillaries, mildly disturbed capillary architecture, and moderate capillary loss; the “late” type was characterized by severe capillary loss with extensive vascular desertification, and disruption of normal capillary architecture. NVC parameters to be measured were capillary density (number of capillaries per 1 mm in the distal row of each finger), giant capillaries (homogeneously enlarged capillaries >50 μm), enlarged capillaries (>20 μm and ≤ 50 μm), micro-bleeding, oedema, avascular areas (the normal range adopted was 9 capillaries per linear millimeter), ramified capillaries, bushy and tortuous capillaries. Capillary's density was evaluated in the distal row of each finger, based on the number of capillaries per 1 mm, and the mean capillaroscopic skin ulcer risk index (CSURI) was automatically calculated with software image analysis.

### Statistical Analysis

Statistical analysis was performed with Statistical Package for Social Sciences 22 (SPSS Inc, Chicago, IL, USA). Continuous variables were expressed as mean values ± standard deviation (SD) or median [interquartile range] according to the normality of distribution tested with the Kolmogorov-Smirnov or the Shapiro-Wilk test. Categorical variables were presented as absolute frequencies and percentages (*n*, %). Comparisons for continuous variables were performed with the student's *t*-test or the Mann-Whitney *U* test, according to the normality of the distribution. Multiple comparison analysis was performed with the ANOVA or the Kruskal-Wallis tests, according to normality. Chi-square or Fishers exact test was used for comparisons of categorical variables. Uni- and multivariable linear regression analysis was performed to explore the parameters possibly associated with the number of avascular areas. We first tested for univariate associations and included in the multivariable model only variables with associations of *p* < 0.2 in univariate analysis. We report β-coefficients with 95% confidence intervals (CI). *P* < 0.05 (two-tailed) were considered statistically significant for all comparisons.

## Results

### Patient Characteristics

Table 1 depicts demographic, anthropometric, clinical, and laboratory characteristics of the study population. In total, 64 Caucasian patients with SSc (95.3% women) with mean age 57.54 ± 12.99 years were included in the study. The number of capillaries per mm were 6 [4], avascular areas were 2 [3] and CSURI index 3.25 [6.57].

**Table 1:** Baseline characteristics of the study participants.

**Parameters**	**Value**
N	64
Age (years)	57.54 ± 12.99
**Gender**	
Men, *n* (%)	3 (4.7)
Women, *n* (%)	61 (95.3)
Weight (kg)	64.25 ± 10.38
Height (cm)	162 [80]
BMI (m^2^)	24.54 ± 3.87
Pulmonary fibrosis *n*, (%)	23 (35.94)
Pulmonary hypertension *n*, (%)	12 (18.75)
Esophageal involvement *n*, (%)	26 (40.63)
ESR (mm/h)	16 [17.5]
CRP (mg/dl)	0.76 [4.34]
Hemoglobin (gr/dl)	12.75 ± 1.14
Uric acid (mg/dl)	4.03 [2.13]
Ur (mg/dl)	33 [15.88]
Cr (mg/dl)	0.80 [0.26]
Potassium (mEq/L)	4.21 [0.75]
Sodium (mEq/l)	140 [2.3]
TChol (mg/dL)	194.86 ± 42.91
LDL(mg/dL)	117.61 ± 38.36
Tgl (mg/dL)	115 [56]
SBP (mmHg)	128.94 ± 19.8
DBP (mmHg)	76.72 ± 9.2
GFR (ml/min/1.73 m^2^)	82.6 ± 24.07
ANA, *n* (%)	62 (96.88)
ACA, *n* (%)	19 (29.69)
Scl-70, *n* (%)	25 (39.01)
Febuxostat, *n* (%)	0 (0)
Allopurinol, *n* (%)	1 (1.56)
Omeprazole, *n* (%)	7 (10.94)
Losartan, *n* (%)	4 (6.25)

*ESR, Erythrocyte sedimentation rate; CRP, C-Reactive Protein; GFR, Glomerular Filtration Rate; SBP, systolic blood pressure; DBP, diastolic blood pressure; ANA, Antinuclear antibodies; ACA, Anti-centromere antibodies; Scl-70, Anti–Scl-70 antibodies*.

### Uric Acid and NVC Measurements

The correlation of UA, urea, creatinine, and GFR with the parameters of NVC is presented in Table 2. Serum UA levels were significantly associated with the number of avascular areas (*r* = 0.290; *p* = 0.020), whereas no correlation was shown for the eGFR (*r* = −0.065; *p* = 0.609). All the other NVC parameters measured showed no correlation with the levels of UA. There was no significant correlation with CSURI (*r* = −0.041; *p* = 0.749).

**Table 2:** Correlation of urea, creatinine, GFR, and UR with NVC parameters.

		**Ur**	**Cr**	**GFR**	**UA**
Capillaries	r	−0.023	−0.127	0.087	−0.157
	p	0.859	0.318	0.492	0.216
Avascular	r	0.060	0.089	−0.065	0.290
	p	0.638	0.482	0.609	0.020
Edema	r	0.144	−0.084	0.051	−0.211
	p	0.255	0.509	0.691	0.094
Microbleeding	r	0.141	−0.069	0.011	0.094
	p	0.267	0.586	0.931	0.460
Enlarged	r	0.062	−0.187	0.198	−0.052
	p	0.624	0.139	0.116	0.681
Giant	r	0.087	0.083	−0.093	−0.037
	p	0.495	0.512	0.464	0.770
Ramified	r	0.036	0.182	−0.155	0.129
	p	0.778	0.150	0.220	0.312
Bushy	r	0.066	0.059	−0.040	0.139
	p	0.604	0.644	0.756	0.272
Tortous	r	−0.004	0.009	−0.051	0.137
	p	0.976	0.942	0.690	0.282
CSURI	r	0.019	0.071	−0.094	−0.041
	p	0.882	0.579	0.462	0.749

*GFR, Glomerular Filtration Rate; UA, uric acid; CSURI, skin ulcer risk index*.

**Figure 1:**
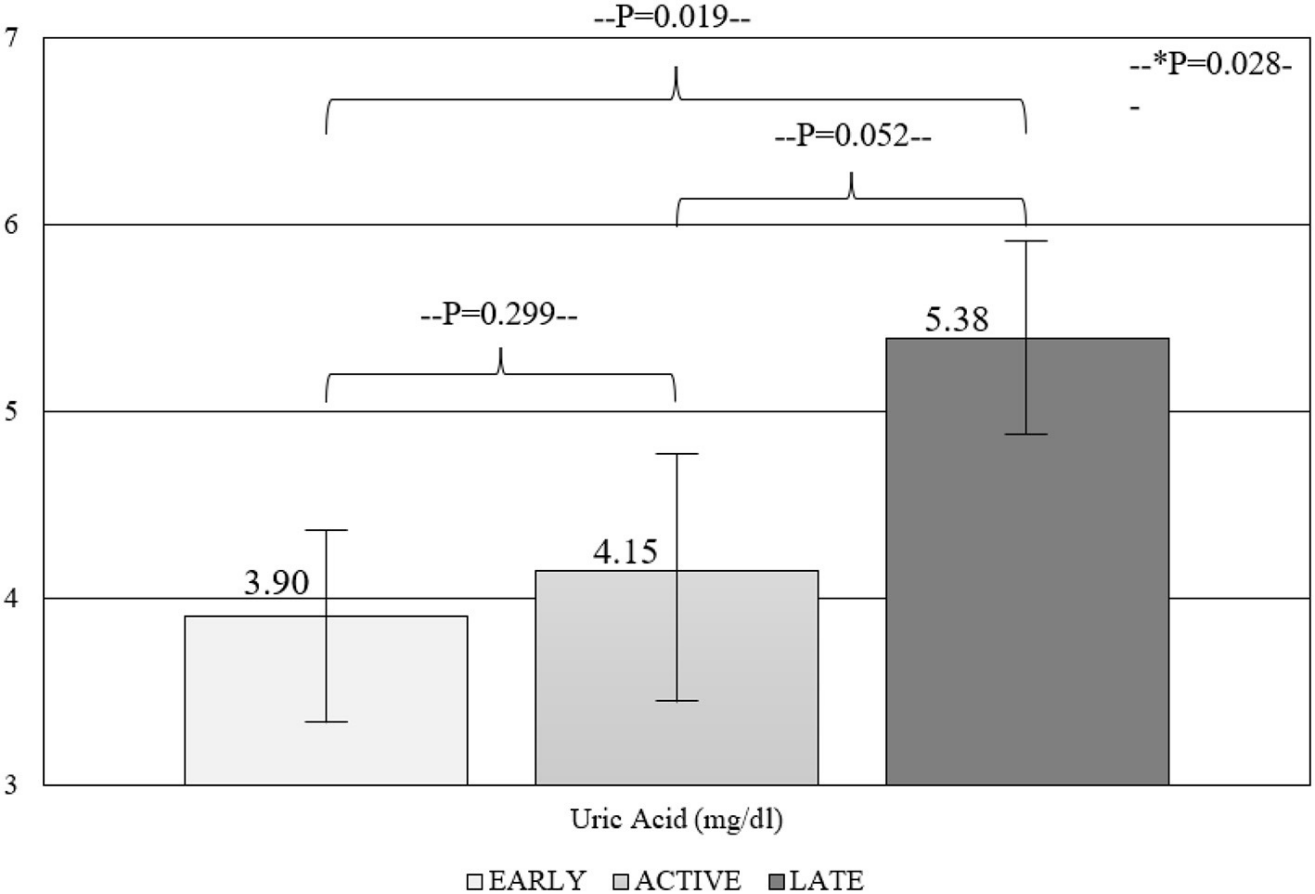
Comparison of UA in the three NVC patterns.

Within-groups comparisons revealed a significant trend of UA levels in the capillaroscopy patterns reflective of progressive micro-vasculopathy (3.90 ± 1.52 vs. 4.15 ± 0.98 vs. 5.38 ± 2.26; for early, active, and late patterns, respectively, *p* = 0.028) (Figure 1). Similarly, the comparison between different NVC pattern groups demonstrated higher UA levels in patients with late compared to early and active patterns (*p* = 0.019 and *p* = 0.052), while the degree of NVC changes was not associated with creatinine or eGFR levels.

### Parameters Associated With Avascular Areas in NVC

In order to identify possible factors associated with the avascular areas in NVC, we have performed a uni- and multivariable linear regression analysis with a number of avascular areas being the dependent variable and several demographic, anthropometric, medical history, and laboratory parameters as the independent variables. As shown in Table 3, in multivariable analysis male gender (β = 3.049; 95% CI = 0.997–5.101) and UA (β = 0.352; 95% CI = 0.117–0.588) were shown to be independently associated with the number of avascular areas, whereas smoking was not.

**Table 3:** Uni- and multivariable linear regression analysis of parameters possibly associated with the number of avascular areas as evidenced with the capillaroscopy.

**Parameter**	**Univariate analysis**				**Multivariable analysis**			
	**Estimate**	**Std error**	**95% CI**	***P* value**	**Estimate**	**Std error**	**95% CI**	***P* value**
Male gender	3.049	1.027	0.997–5.101	**0.004**	2.492	1.011	0.469–4.515	**0.017**
Age	0.016	0.018	−0.020–0.051	0.388				
BMI	−0.055	0.060	−0.175–0.065	0.362				
Smoking	−0.826	0.505	−1.836–0.184	0.107	−0.312	0.491	−1.295–0.671	0.528
Hypertension	0.373	0.498	−0.623–1.368	0.457				
eGFR	−0.003	0.010	−0.022–0.016	0.762				
UA	0.352	0.118	0.117–0.588	**0.004**	0.272	0.121	0.030–0.514	**0.028**
CRP	0.010	0.008	−0.007–0.026	0.240				
SBP	−0.008	0.012	−0.031–0.016	0.508				
DBP	−0.016	0.025	−0.066–0.035	0.540				

*GFR, Glomerular Filtration Rate; UA, uric acid; CRP, C-Reactive Protein; SBP, systolic blood pressure; DBP, diastolic blood pressure. Bold letters represent p < 0.05*.

## Discussion

The main finding of our study is the positive correlation between NVC structural alterations and serum UA concentrations in patients with SSc. In particular advancing stages of SSc-related microangiopathy as determined by both NVC “scleroderma patterns” and the number of avascular areas are associated with higher UA levels, indicating UA as a potential biomarker of peripheral vascular damage in SSc.

This possibility may reflect biologically relevant metabolic procedures involved in the pathogenesis and progression of SSc-related microvasculopathy. For example degradation of UA by the oxidative action of the enzyme xanthine oxidoreductase leads to the production of reactive oxygen species and the initiation of several detrimental procedures such as increased cytokine production, inflammation, and endothelial activation all of which contribute to vascular injury (18). Taking into account that SSc is regarded as a disease of increased oxidative stress (19), upregulation of oxygen free radicals and low antioxidant defense capacity driven by increased UA levels (20) may play a crucial role in the pathophysiology of microangiopathy.

Thus, it is not surprising that previous reports have demonstrated significant associations between high UA levels and various aspects of SSc vasculopathy. In fact elevated UA levels do not only confer a diagnostic value in the identification of SSc individuals at early, asymptomatic stages of PAH as indicated by DETECT study (21), but they also serve as a useful biochemical tool for the assessment of PAH severity (22) and outcomes as they appear to be associated with survival in these patients (23). Besides pulmonary microvasculature, high UA levels were independently associated with the occurrence of digital ulcers in a cross-sectional study including 71 persons with SSc (9) providing another link between peripheral microangiopathy and oxidative stress in this condition. In addition, UA levels have been linked with the extent of renal microvasculopathy in SSc defined as increased intrarenal stiffness (10).

Our findings are in line with a previous small study (10) and confirm the positive correlation between UA levels and peripheral microvascular damage in SSc individuals. Furthermore, we demonstrated that UA levels are increasing in correlation with progressive stages of SSc microangiopathy, particularly in the presence of late (worse) NVC pattern suggesting that UA may contribute to the evolution of microcirculatory abnormalities in SSc. However, the cross-sectional design of all the aforementioned studies—including the current one—does not allow the establishment of any temporal relationships between UA and rarefaction of digital arteries in SSc. Whether such observations indicate a reverse causality, implying that elevated UA levels may not have a direct crucial effect on endothelial derangement and subsequent vascular injury, but they rather represent an easily measurable byproduct of oxidative stress remains to be determined in longitudinal or experimental studies.

On the other hand, the relationship between high UA and various markers of microvascular dysfunction in different vascular beds namely retinal arteriolar narrowing (24), microalbuminuria (25) and coronary flow reserve (26) has been described in previous studies especially in patients with higher cardiovascular risk profile. Considering the increased rate of cardiovascular events such as stoke and myocardial infarction among SSc patients (27) and the well-established contribution of hyperuricemia to the occurrence and development of cardiovascular disease in both rheumatic diseases and general population (28–30), the results of our study provide further insights in the complex association between micro- and macro vascular involvement in SSc. A growing amount of evidence point toward significant correlations between higher grades of SSc microangiopathy and indices of cardiovascular disease such as arterial stiffness (31) and cardiomyopathy (13, 32). Interestingly enough, a recent study by our group indicated that worsening phases of NVC patterns were associated with higher cardiovascular risk scores (33) suggesting that excessive capillary loss and macrovascular endothelial dysfunction may be closely interrelated and promote cardiovascular disease in this population. In this regard, the demonstrated association between progressive microvascular injury and higher UA levels may indicate UA as a marker of generalized, widespread vasculopathy in SSc including both micro- and microvasculature.

The main limitation of our study is the single-center, cross-sectional design which precludes any causal relationships between UA levels and vasculopathy as discussed above. However, NVC was performed in all fingers except thumbs and the acquisition of two adjacent images from each finger according to the updated European League against Rheumatism (EULAR) recommendations (34). We took particular care to include individuals with SSc, with a broad spectrum of disease-related visceral involvement as well as comorbidities representative of a real-life population with SSc. To the best of our knowledge, this is the largest study to investigate the association between UA and microvascular injury by detailed qualitative and semi-quantitative assessment based on a validated algorithm.

In conclusion, serum levels of UA are significantly associated with progressive micro-vasculopathy based on the qualitative NVC pattern. These results provide evidence that UA may be a significant mediator of the microvascular damage in these patients.

## Data Availability Statement

The raw data supporting the conclusions of this article will be made available by the authors, without undue reservation.

## Ethics Statement

The studies involving human participants were reviewed and approved by Ethics Committee of School of Medicine, Aristotle University Thessaloniki, Greece. The patients/participants provided their written informed consent to participate in this study.

## Author Contributions

EP collected the data and drafted the paper with support from TD. SS contributed to the perception of the study and the editing of the paper. ET collected the data. AM verified the analytical methods and performed the analysis. GK and AG were involved in planning and supervised the work and critically reviewed the paper. TD supervised the project and contributed to drafting the paper. All authors discussed the results and contributed to the final manuscript.

## Conflict of Interest

The authors declare that the research was conducted in the absence of any commercial or financial relationships that could be construed as a potential conflict of interest.

## Publisher's Note

All claims expressed in this article are solely those of the authors and do not necessarily represent those of their affiliated organizations, or those of the publisher, the editors and the reviewers. Any product that may be evaluated in this article, or claim that may be made by its manufacturer, is not guaranteed or endorsed by the publisher.
